# LGBTQ+ INCLUSIVITY TRAINING AND EDUCATION FOR SKILLED NURSING FACILITIES

**DOI:** 10.1093/geroni/igad104.3450

**Published:** 2023-12-21

**Authors:** Jennifer May, Alexis Domeracki, Perisa Ashar, Foxx Hart, Jason Wheeler, Glaucia Salgado, Melanie Wang

**Affiliations:** University of South Carolina, Columbia, South Carolina, United States; Duke School of Medicine, Durham, North Carolina, United States; Duke University, Durham, North Carolina, United States; Duke University, Durham, North Carolina, United States; Duke PHMO, Durham, North Carolina, United States; Duke Clergy Heath, Durham, North Carolina, United States; Duke University, Durham, North Carolina, United States

## Abstract

The population of older adults is growing in the United States, leading to greater need for specialized care offered by skilled nursing and long-term care facilities. Among older adults that require specialized health care, lesbian, gay, bisexual, transgender, queer, and other sexual and gender minority (LGBTQ+) older adults are more likely to experience health disparities and health care barriers due to lifelong discrimination and exclusion. Healthcare workers within skilled nursing facilities (SNF) have expressed they do not feel prepared to care for LGBTQ+ older adults. The aim of this research was to develop the LGBTQ+ Inclusivity Training and Education (LITE) toolkit to provide information to healthcare workers, staff, residents, and their families in the SNF environment. Phase 1 used a community engaged approach to develop the LITE toolkit through engagement with an LGBTQ+ Community Advisory Board. Phase 2 was the implementation and evaluation of the LITE toolkit with 25 SNFs throughout North Carolina. The majority of SNF healthcare workers and staff (N=20) who responded to the survey indicated that the LITE toolkit was “easy to use,” they were satisfied with the contents, and that it would improve the way they care for patients. Providing LGBTQ+ focused education for members of the SNF community addresses the need for healthcare worker and staff training, thereby contributing to equitable care and inclusive environments for the LGBTQ+ older adult community. Additional work focused on understanding the facilitators and barriers to using the LITE toolkit in the SNF setting is needed.

